# Sentinel-Site-Based Surveillance of *Mycobacterium tuberculosis* Drug Resistance and Epidemiology in Sichuan, China

**DOI:** 10.3390/antibiotics14070625

**Published:** 2025-06-20

**Authors:** Yiting Wang, Chunfa Liu, Bing Zhao, Xichao Ou, Hui Xia, Yuanyuan Song, Yang Zheng, Yang Zhou, Ruida Xing, Yanlin Zhao, Huiwen Zheng

**Affiliations:** 1 Institute for Immunization and Prevention, Beijing Center for Disease Control and Prevention, Beijing Academy for Preventive Medicine, Beijing Institute of Tuberculosis Control Research and Prevention, Beijing 100013, China; 2 National Tuberculosis Reference Laboratory, Chinese Center for Disease Control and Prevention, Beijing 102200, China; 3 Department of Animal Science and Technology, Beijing University of Agriculture, Beijing 100096, China; 4 Laboratory of Respiratory Diseases, Beijing Key Laboratory of Pediatric Respiratory Infection Diseases, Key Laboratory of Major Diseases in Children, Ministry of Education, National Clinical Research Center for Respiratory Diseases, National Center for Children’s Health, Beijing Pediatric Research Institute, Beijing Children’s Hospital, Capital Medical University, Beijing 100045, China

**Keywords:** tuberculosis, whole-genome sequencing, drug resistance, transmission, risk

## Abstract

Objectives: To investigate epidemiological/drug-resistance characteristics and identify potential factors related to drug-resistant and clustered tuberculosis in Sichuan. Methods: A total of 295 *Mycobacterium tuberculosis* (MTB) isolates were collected from surveillance sites in Sichuan from 2019 to 2021. The minimum inhibitory concentrations (MICs) of 12 anti-TB drugs were acquired using the broth microdilution method, followed by whole-genome sequencing (WGS) analysis. Results: Of 268 MTB isolates with both WGS and drug-susceptibility testing (DST) results, 159 (59.3%, 159/268) strains belonged to the Beijing lineage (L2). Isoniazid had the highest resistance rate (15.3%, 41/268), followed by rifampicin (9.3%, 25/268). The sensitivity of WGS to predict drug resistance varied from 75% to 97.6%, and the specificity was above 96.0% for all. *rpoB* Ser450Leu (41.7%, 10/24) and *katG* Ser315Thr (70%, 28/40) were the most frequent mutations in rifampicin and isoniazid resistance isolates, respectively. The clustering rate in Sichuan was 9.3% (25/268), and patients ≤ 24 years old (aOR = 11.697; 95% CI: 0.817–167.463) had a greater risk of clustering. Conclusions: Our findings prove that WGS is a promising tool for predicting drug resistance to isoniazid, rifampicin, ethambutol, moxifloxacin and levofloxacin in Sichuan. The higher resistance rate to isoniazid emphasizes the urgent need for susceptibility testing surveillance and application management. Improving the diagnosis, treatment and management of patients ≤ 24 years old may reduce the transmission of MTB in Sichuan.

## 1. Introduction

With an estimated 1.25 million deaths globally, tuberculosis (TB) returned to the top single cause of death in 2023, threatening global public health [1]. Though substantial efforts have been made, China still ranked the third largest TB burden in indices globally [2]. In addition, the continued high incidence of drug-resistant TB hampers its prevention and control, with an estimated 29,000 patients with multidrug/rifampicin-resistant TB (MDR/RR-TB) in China [3]. Compared to drug-susceptible TB, the relative transmission rate is higher for MDR-TB, which is an important driving force for drug-resistant TB [4,5,6]. To develop a national response to drug-resistant tuberculosis, the Chinese Center for Disease Control and Prevention (CDC) has conducted national drug-resistant surveillance (DRS) annually since 2007 [7]. But there are significant regional differences in the prevalence of TB and drug-resistance characteristics [8,9,10,11]. Sichuan, located in southwest China, is a mountainous area and comprises a large migrant population, which has a top-ranked TB burden [12,13,14,15]. So, high-resolution surveillance data is needed to monitor strain transmission and resistance, allowing the implementation of targeted TB control and treatment measures.

Phenotypic drug susceptibility testing (pDST), the conventional culture-based gold standard for diagnosing drug-resistant tuberculosis, faces significant limitations including prolonged turnaround time (4–6 weeks), restricted drug coverage and stringent biosafety requirements [16,17]. Molecular techniques, such as GeneXpert MTB/RIF (Cepheid) and GenoType MTBDRplus (Hain Lifescience), have emerged as a solution to these limitations and are now widely employed for rapidly detecting mutations in genes associated with resistance. However, these molecular approaches are limited to only predefined resistance-associated mutations, potentially missing novel variants, and lack sensitivity in detecting heteroresistance [18,19,20].

As a rapid, reliable and increasingly affordable technology, the whole-genome sequencing (WGS) approach has been used to investigate TB transmission dynamics and outbreaks and explore patterns of resistance [21,22,23]. Compared to conventional genotyping methods, WGS has a higher discriminatory power to trace infection sources and transmission networks [24]. In addition, WGS can predict antimicrobial susceptibility profiles based on known mutations of resistance, allowing prompt, appropriate initiation of treatment and monitoring acquisition of drug resistance [21,25]. Here, we performed WGS and phenotypic drug-susceptibility testing (DST) against 12 anti-TB drugs for *Mycobacterium tuberculosis* (MTB) isolates across Southwest China to investigate the epidemiological, drug-resistance phenotypic and genotypic characteristics and identify the factors related to drug-resistant and clustered tuberculosis.

## 2. Results

### 2.1. Demographic and Clinical Characteristics

To elucidate the epidemiological profile of tuberculosis patients in Sichuan province, we characterized the baseline demographic and clinical features of the enrolled cases. Among 268 patients, 69.8% (187/268) were male, 32.5% (87/268) were aged between 45 and 64 years, and 73.1% (196/268) were from rural areas. More than half (57.5%, 154/268) were farmers, and 92.9% (249/268) were Han. Overall, 7.1% (19/268) had diabetes, 10.4% (28/268) had TB exposure history, and 14.2% (38/268) cases had received previous treatment (Table 1).

**Figure 1:**
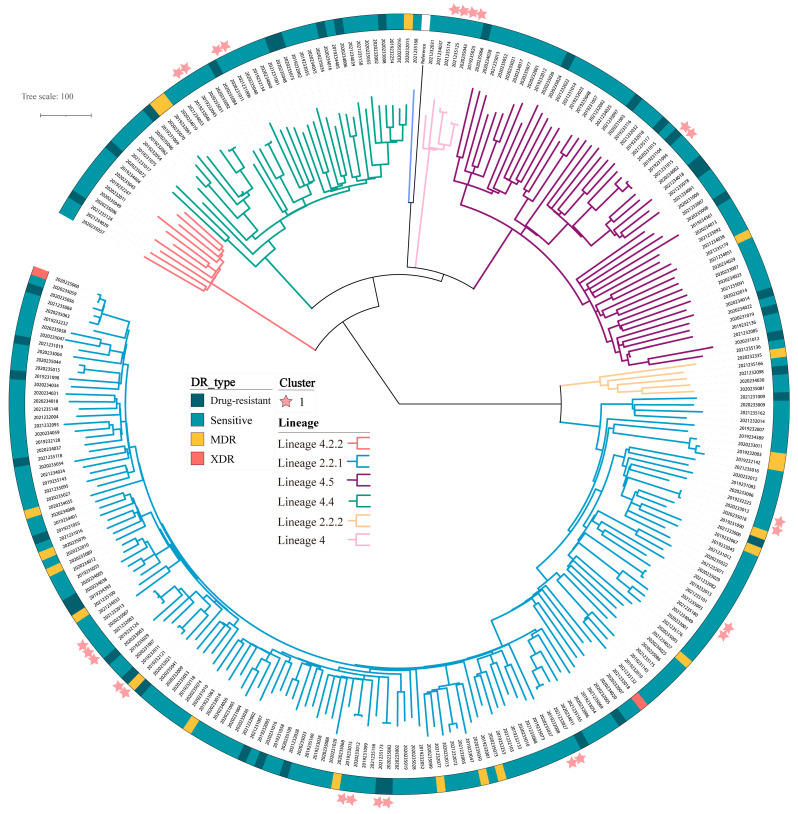
Phylogeny, clustering and resistance profile of 268 *Mycobacterium tuberculosis* strains. Drug-resistant types are represented by different colors in strips around the tree. Dark blue indicates drug-resistant strains, cyan represents sensitive strains, orange corresponds to multidrug resistant (MDR) strains, and red stands for extensively drug-resistant (XDR) strains. Lineages: different lineages and sublineages are distinguished by branches of various colors. Clusters: the clustered strains are symbolized by stars. Each branch in the tree represents the evolutionary relationships.

To characterize genomic polymorphisms, evolutionary relationships and transmission dynamics among *M. tuberculosis* strains, we performed whole-genome sequencing-based phylogenetic analysis. The results showed that 159 (59.3%, 159/268) strains belonged to the Beijing lineage (L2), including 2.5% (4/159) of the L2.2.2 sublineage and 93.1% (148/159) of the L2.2.1 sublineage. In addition, 109 (40.7%, 109/268) isolates belonged to the Euro-American lineage (L4), including 10.1% (11/109) of sublineage 4.2.2, 33% (36/109) of sublineage 4.4.2 and 49.5% of sublineage 4.5 (54/109) (Figure 1).

### 2.2. Drug-Resistance Characteristics

**Figure 2:**
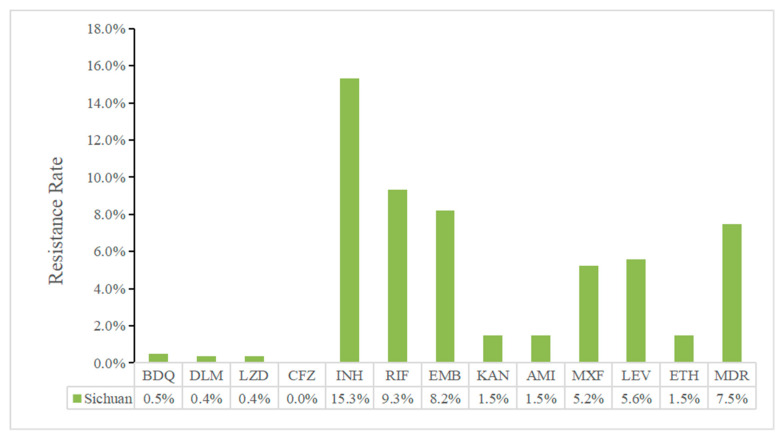
Drug-resistance profile of all 268 clinical MTB isolates according to culture-based DST results.

**Figure 3:**
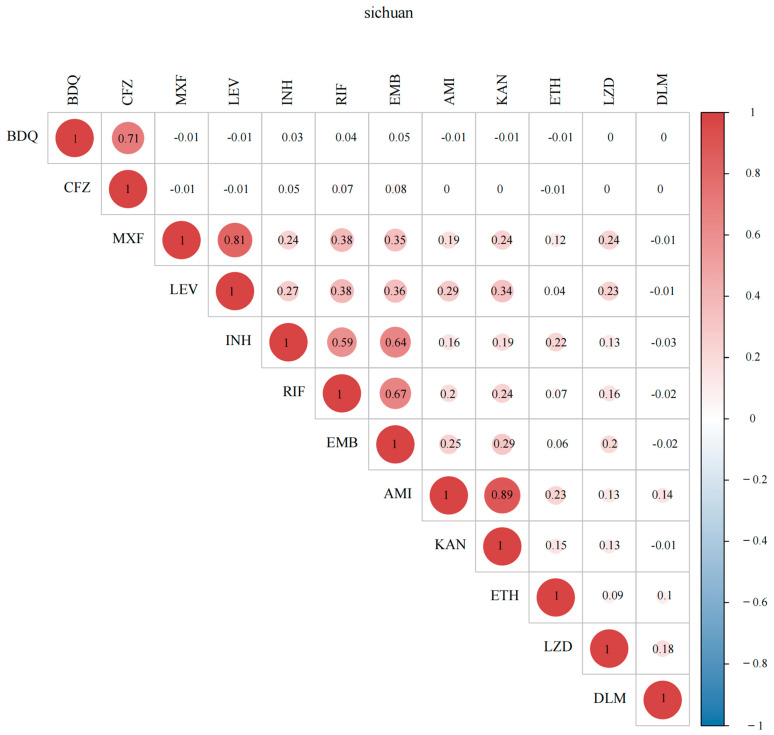
Resistance correlation matrix of 268 *M. tuberculosis* strains of Sichuan province.

Analyzing *M. tuberculosis* drug-resistance profiles is crucial for guiding targeted TB prevention, treatment optimization and transmission control. Of all the 268 isolates, INH had the highest resistance rate (15.3%, 41/268), followed by RIF (9.3%, 25/268), EMB (8.2%, 22/268), LEV (5.6%, 15/268) and MXF (5.2%, 14/268) (Figure 2). And 7.8% (21/268) strains showed isoniazid-resistance and rifampicin susceptibility (Hr-Rs). A total of 7.5% (20/268) of the patients were MDR-TB, with 6.3% (17/268) in new TB cases and 1.1% (3/268) in retreated TB cases. A high frequency of co-resistance was observed between AMI and KAN, MXF and LEV, BDQ and CFZ, RIF and EMB, EMB and INH and RIF and INH (r > 0.5), and a moderate frequency of co-resistance was exhibited between EMB and FQs and RIF and FQs (0.3 < r < 0.5) (Figure 3).

The size of the circle and the number represent the correlation coefficient r (−1, 1). When r > 0, it indicates a positive correlation between variables. The larger the |r| value, the stronger the correlation. Generally, 0.8 ≤ |r| ≤ 1 is defined as extremely strong correlation, 0.6 ≤ |r| < 0.8 as strong correlation, 0.2 ≤ |r| < 0.4 as moderate correlation and |r| < 0.2 as no correlation.

### 2.3. Genetic Determinants of Resistance

Due to the small number of resistant isolates for BDQ, DLM, LZD and CFZ, we excluded them from evaluating the ability of WGS to predict drug resistance. For the other drugs, the sensitivity of the WGS varied from 75% to 97.6%, the specificity was all above 96% (Table 2). Of 25 phenotypic RR-TB strains, 24 (96%, 24/25) isolates had mutations in the *rpoB* gene, with *rpoB* Ser450Leu as the most frequent mutation (41.7%, 10/24). Among the 40 (97.6%, 40/41) isolates that had mutations with INH resistance, the most common mutations were *katG* Ser315Thr (70%, 28/40). Ethambutol resistance mutations (86.4%, 19/22) occurred majorly in *embB* Met306Ile (21.1%, 4/19) and Met306Val (21.1%, 4/19), and *gyrA* Ala90Val (33.3%, 4/12) was the most frequent mutation in FQ-resistant isolates (80.0%, 12/15). The *rrs* A1401G (100%, 3/3) was the most common in aminoglycoside resistance mutations (75%, 3/4), and *fabG1* C-15T (100%, 3/3) majorly occurred mainly in ETH resistance mutations (75%, 3/4). No mutations were identified in BDQ, DLM, LZD and CFZ resistance isolates (Table S2).

### 2.4. Transmission of MTB and Associated Risk Factors

Investigating MTB transmission dynamics and underlying risk determinants is essential to elucidate epidemic patterns, identify modifiable drivers and develop targeted interventions to disrupt transmission chains. A total of 25 isolates (9.3%, 25/268) were clustered into 12 genomic clusters, with 2–3 strains in each cluster. Of 12 genomic clusters, 3 (25.0%, 3/12) clusters were collected from the different surveillance sites (Supplementary Figure S1). The multivariable logistic regression analysis showed that patients ≤ 24 years old (aOR = 11.697; 95% CI: 0.817–167.463) had a greater risk of clustering (Table 3).

## 3. Discussion

Analyzing the epidemiological, drug-resistance and transmission characteristics of *Mycobacterium tuberculosis* isolate-related localized data is essential to implement more efficient TB prevention and control strategies. According to previous reports, the Beijing lineage was the predominant lineage in China and varied from 44% to 93% across all provinces [26,27]. We found that 59.3% of isolates were assigned to lineage 2, lower than that of another report in Sichuan (82.01%) [28]. The observed variation can be attributed to the fact that the latter study specifically focused on MDR/RR-TB isolates, and the establishment of MDR epidemics was associated with lineage 2 [29,30,31]. Similar to a previous report [27], the proportion of lineage 4 was higher than that of other regions in China [32,33], indicating that a foreign origin from overseas is likely for these strains.

According to a previous report, the development of isoniazid resistance is a common first step in the evolution of MDR-TB [29]. In the present study, the highest resistance rate (15.3%) was observed in INH, and 7.8% of strains showed Hr-Rs, slightly higher than previously reported (3.9–4.6%) in eastern China [30,34]. Considering the high failure rates (18–44%) in patients with mono-resistance to INH and poor treatment outcomes for Hr-Rs patients with standard first-line therapy, more attention should be paid to the rapid susceptibility testing of INH [31,35]. Moreover, prevention of drug-resistant tuberculosis, especially MDR-TB, is an essential part of tuberculosis-control programs. We found that 6.3% of new patients were MDR-TB, higher than the national level (2.1%) in 2023 [3], indicating that the MDR-TB status in Sichuan remains serious. The proportion of MDR-TB being different between regions may be due to differences in the quality of the local TB control programs. The low proportion of drug resistance to BDQ, DLM, LZD and CFZ provides support for the implementation of BPaL-based regimens in Sichuan. We also found that there is a certain degree of correlation between drugs, indicating that there may exist shared resistance mechanisms or different drug combinations/use preferences.

WGS has been proven a powerful tool for predicting the drug resistance of *Mycobacterium tuberculosis*, especially for first-line drugs [36]. Consistent with a previous report [37], WGS is a promising approach to predict resistance to INH, RIF, EMB, MXF and LEV with a sensitivity of above 80%. The lower sensitivity of WGS in predicting AMI (75.0%), KAN (75.0%) and ETH (75%) resistance indicated that some non-specific mechanisms were associated with the resistance, such as efflux pumps.

Similar to a previous report [38], this study also showed that 96% of RIF-resistant isolates harbored mutations in the *rpoB* gene. And the most common mutation was observed in Ser450Leu (49.4%), which has been considered with high levels of RIF resistance [37,39]. The major mutation observed in INH resistance was in *katG* Ser315Thr (70.0%), inducing high-level resistance [37]. A previous study revealed that *embB* Met306Val was the most frequent mutation in EMB-resistant isolates [37,40], but Met306Ile was predominant in this study. The most frequent FQ-resistance-conferring mutation was *gyrA* Ala90Val (33.3%), which is different from a previous study with *gyrA* Asp94Gly being predominant [37,40]. These differences may be attributed to the transmission of resistant strains or region-specific evolutionary pressures.

We found that the clustering rate of isolates was 9.3%, lower than the national clustering rate (23%) and the proportions reported in Shanghai (31%) [5,41], reflecting that the local transmission of TB was low in Sichuan. Many factors could affect TB transmission, including samples and the methods adopted, host factors, bacterial factors and local TB control programs. Our study showed that patients ≤ 24 years old had a high risk of clustering, who usually had greater learning pressure and lacked exercise, and the congregated environment may create conditions for the transmission of TB. So timely detection of adolescent patients and blocking the transmission of TB in school are of great significance for TB control.

## 4. Conclusions

In conclusion, our findings prove that WGS is a promising tool for predicting drug resistance to INH, RIF, EMB, MXF and LEV in Sichuan. The higher resistance rate to INH emphasizes the urgent need for susceptibility testing surveillance and application management. Improving the diagnosis, treatment and management of patients ≤ 24 years old may reduce the transmission of MTB in Sichuan.

## 5. Methods

### 5.1. Isolate Collection

**Figure 4:**
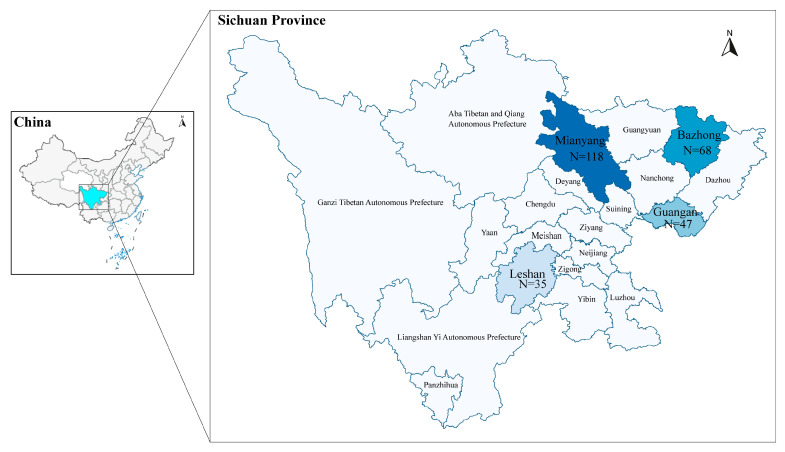
Map of the distribution of the tuberculosis patients whose isolates of *Mycobacterium tuberculosis* were included in the study.

**Figure 5:**
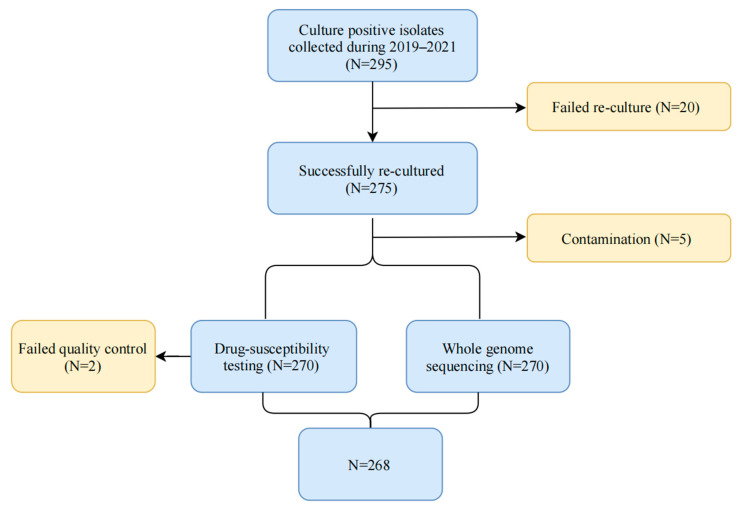
Flow chart of the isolates included in the study.

The 295 MTB isolates were obtained from TB patients from Sichuan provinces, corresponding to 4 regions (Mianyang, Bazhong, Guangan and Leshan) included in the Chinese DRS Program between 2019 and 2021 (Figure 4). The surveillance site selection referred to the first national survey of drug resistance [7]. After excluding 27 samples (20 failed re-culture, 5 failed contamination, and 2 failed testing), 268 MTB isolates with both WGS and DST results were available (Figure 5).

### 5.2. Drug Susceptibility Testing

Phenotypic DST for a panel of 12 anti-TB drugs (bedaquiline [BDQ], delamanid [DLM], linezolid [LZD], clofazimine [CFZ], isoniazid [INH], rifampicin [RIF], ethambutol [EMB], kanamycin [KAN], amikacin [AMI], ethionamide [ETH], moxifloxacin [MXF] and levofloxacin [LEV]) was performed using the minimum inhibitory concentration (MIC) method by a commercial dry plate (Thermo fisher, UKMYC5 and UKMYC6 plate), as previously described [25]. Bacterial colonies of *Mycobacterium tuberculosis* in the logarithmic growth phase (approximately 14 days) were scraped into a sterile grinding tube containing 2–3 mL of saline(Solarbio, Beijing, China). The bacterial suspension was homogenized using an ultrasonic disperser and adjusted to a turbidity of 0.5 McFarland units. After thorough mixing, 100 µL of the suspension was added to 10 mL of Middlebrook 7H9 broth (OXOID, USA) supplemented with 10% oleic acid-albumin-dextrose-catalase (OADC) (BD, Middlebrook, Knoxville, TN, USA). The bacterial inoculum was then uniformly dispensed into a commercial drug susceptibility testing microplate using an automated liquid handler (100 µL per well). The microplate was incubated at 37°C with 5% CO_2_ for 10–14 days. The MTB H37Rv (ATCC 27294) strain was used to quality control (QC) all tests. The breakpoint for each drug is shown in Supplementary Material (Table S1). MIC was defined as the lowest concentration without obvious visible bacterial growth compared with positive controls. MDR isolates were defined as MTB isolates resistant to at least INH and RIF, the key first-line drugs, confirmed by the proportional method on the Löwenstein–Jensen medium [42].

### 5.3. Genomic DNA Preparation

Genomic DNA was extracted using the cetyltrimethylammonium bromide (CTAB) method with the following protocol: Fresh *Mycobacterium tuberculosis* cultures (50–100 mg) were transferred to screw-cap tubes, inactivated at 80 °C for 30 min and centrifuged at 12,000 rpm for 5 min (supernatant discarded). The pellet was resuspended in 400 μL TE buffer (Solarbio, Beijing, China) by pipetting, followed by the addition of 50 μL lysozyme (10 mg/mL) (Amresco, Solon, OH, USA)and overnight incubation at 37 °C (16–20 h). Subsequently, 70 μL of 10% SDS (Merck, Darmstadt, Germany)and 5 μL proteinase K (20 mg/mL) (Solarbio, Beijing, China) were added, mixed by vortexing and incubated at 65 °C in a metal bath for 10 min. After adding 100 μL of 5 M NaCl and a 100 μL CTAB/NaCl solution, the mixture was inverted until milky white and incubated at 65 °C for 10 min. DNA was extracted with 750 μL chloroform:isoamyl alcohol (24:1), mixed by inversion and centrifuged (12,000 rpm, 5 min). The aqueous phase was transferred to a new tube, mixed with 0.6 volumes of ice-cold isopropanol and stored at −20 °C for 30 min for DNA precipitation. Following visible white precipitate formation, the sample was centrifuged (12,000 rpm, 15 min) and washed with 1 mL ice-cold 70% ethanol (12,000 rpm, 5 min). The DNA pellet was air-dried at 37 °C for 10 min and finally dissolved in 50 μL TE buffer. Each extracted DNA was quantified by Qubit 2.0 Fluorometer (Invitrogen, Carlsbad, CA, USA).

### 5.4. Whole-Genome Sequencing Analysis

The qualified DNA was sequenced using the Illumina (San Diego, CA, USA) HiSeq 2000 platform with an expected coverage of 100 times [37,43]. All raw WGS data were processed with the Clockwork pipeline as described previously [44]. Sequences containing genes of the proline–glutamic acid (PE)/proline–proline–glutamic acid (PPE) family, repetitive sequences, phage sequences, mobile genetic elements, insertions and deletions were excluded from the analysis [43]. Sequencing reads corresponding to drug-resistance-related genes were aligned to the reference genome H37Rv (GenBank ID: NC_000962.3). Single-nucleotide polymorphisms (SNPs) were called using SAMtools (V1.21) mpileup and gene annotation generated using snpEff software (version 5.2f). A phylogenetic tree was visualized and modified with iTOL (v 6.4.3) [43]. Isolates with pairwise genetic distances ≤ 12 (SNPs) were defined as a genomic cluster and were considered likely to be consistent with transmission [45]. Genotypic drug-resistance profiles were predicted using a TB-Profiler tool (v4.1.0) [46].

### 5.5. Statistical Analysis

All statistical analysis was performed in the SPSS version 18.0 software (SPSS Inc., Chicago, IL, USA). The categorical variables were presented as numbers and percentages. The logistic regression analysis was used to identify the risk factors associated with RR-TB and clustered TB, expressed as odds ratios (OR) and 95% confidence intervals (CI). Multivariable models were built using “Enter” logistic regression procedures to estimate the adjusted odds ratios (aOR). A *p*-value less than 0.05 was considered statistically significant.

## Figures and Tables

**Table 1:** Demographic information and clinical characteristics of the study population.

Category	Factors	Count (n = 268)	Percentage (%)
Sex			
	male	187	69.8
	female	81	30.2
Residence			
	rural	196	73.1
	urban	72	26.9
Age (year)			
	≤24	44	16.4
	25–44	96	35.8
	45–64	87	32.5
	≥65	41	15.3
Occupation			
	farmer	154	57.5
	employee	21	7.8
	retired	52	19.4
	student	9	3.4
	other	32	11.9
Ethnicity			
	Han	249	92.9
	others	19	7.1
Education			
	uneducated	37	13.8
	primary	92	34.3
	junior	80	29.9
	senior	44	16.4
	college or above	15	5.6
Diabetes			
	Yes	19	7.1
	No	249	92.9
TB exposure			
	Yes	28	10.4
	No	240	89.6
Previous treatment			
	Yes	38	14.2
	No	230	85.8

**Table 2:** Prediction of phenotypes of resistance to drugs.

Drug	WGS Results	Phenotypic DRS		Sensitivity (%)	Specificity (%)	Consistency (%)
		R	S			
Isoniazid	R	40	1	97.6	99.6	99.3
	S	1	226			
Rifampicin	R	24	2	96	99.2	98.9
	S	1	241			
Ethambutol	R	19	2	86.4	99.2	98.1
	S	3	244			
Kanamycin	R	3	0	75	100	99.6
	S	1	264			
Amikacin	R	3	0	75	100	99.6
	S	1	264			
Moxifloxacin	R	12	9	85.7	96.5	95.9
	S	2	245			
Levofloxacin	R	12	9	80	96.4	95.5
	S	3	244			
Ethionamide	R	3	5	75	98.1	97.8
	S	1	259			

WGS—whole-genome sequencing; DRS—drug sensitivity testing; R—resistance; S—susceptibility.

**Table 3:** Univariable and multivariable logistic regression of risk factors for clustered TB.

Category	Factors	Cluster (n = 25)	Non-Cluster (n = 243)	cOR (95% CI)	*p* Value	aOR (95% CI)	*p* Value
Sex							
	male	16 (64%)	171 (70.4%)	1.336 (0.564–3.163)	0.51	1.172 (0.436–3.147)	0.753
	female	9 (36%)	72 (29.6%)	Ref		Ref	
Residence							
	rural	17 (68%)	179 (73.7%)	1.316 (0.542–3.197)	0.544	1.426 (0.465–4.377)	0.535
	urban	8 (32%)	64 (26.3%)	Ref		Ref	
Age							
	≤24	10 (40%)	34 (14%)	11.765 (1.432–96.637)	0.022	11.697 (0.817–167.463)	0.07
	25–44	8 (32%)	88 (36.2%)	3.636 (0.44–30.059)	0.231	5.455 (0.518–57.429)	0.158
	45–64	6 (24%)	81 (33.3%)	2.963 (0.345–25.452)	0.322	3.646 (0.386–34.408)	0.259
	≥65	1 (4%)	40 (16.5%)	Ref		Ref	
Occupation							
	farmer	10 (40%)	144 (59.3%)	0.375 (0.119–1.184)	0.094	0.828 (0.196–3.505)	0.798
	employee	1 (4%)	20 (8.2%)	0.27 (0.029–2.495)	0.248	0.286 (0.028–2.954)	0.294
	retired	5 (20%)	47 (19.3%)	0.574 (0.152–2.165)	0.413	0.887 (0.199–3.951)	0.875
	student	4 (16%)	5 (2.1%)	4.32 (0.851–21.929)	0.078	2.985 (0.461–19.341)	0.251
	other	5 (20%)	27 (11.1%)	Ref		Ref	
Ethnicity							
	Han	24 (96%)	225 (92.6%)	Ref		Ref	
	others	1 (4%)	18 (7.4%)	1.92 (0.245–15.022)	0.534	0.873 (0.098–7.806)	0.904
Education							
	uneducated	1 (4%)	36 (14.8%)	Ref		Ref	
	primary	7 (28%)	85 (35%)	0.181 (0.015–2.162)	0.177	0.435 (0.024–7.963)	0.574
	junior	5 (20%)	75 (30.9%)	0.535 (0.1–2.862)	0.465	1.156 (0.131–10.189)	0.896
	senior	10 (40%)	34 (14%)	0.433(0.076–2.475)	0.347	0.59 (0.077–4.533)	0.612
	college or above	2 (8%)	13 (5.3%)	1.912 (0.368–9.927)	0.441	1.42 (0.222–9.064)	0.711
Diabetes							
	yes	3 (12%)	16 (6.6%)	0.517 (0.14–1.913)	0.323	0.321 (0.065–1.581)	0.163
	no	22 (88%)	227 (93.4%)	Ref		Ref	
TB exposure							
	yes	1 (4%)	27 (11.1%)	3 (0.39–23.072)	0.291	4.668 (0.522–41.771)	0.168
	no	24 (96%)	216 (88.9%)	Ref		Ref	
Previous treatment							
	yes	1 (4%)	37 (15.2%)	4.311 (0.566–32.847)	0.158	2.629 (0.323–21.376)	0.366
	no	24 (96%)	206 (84.8%)	Ref		Ref	
RR-TB							
	yes	1 (4%)	24 (9.9%)	2.63 (0.341–20.314)	0.354	4.77 (0.46–49.481)	0.191
	no	24 (96%)	219 (90.1%)	Ref		Ref	
Lineage							
	lineage 2	14 (56%)	145 (59.7%)	Ref		Ref	
	lineage 4	11 (44%)	98 (40.3%)	0.86 (0.375–1.973)	0.722	0.835 (0.331–2.107)	0.702

cOR—crude odds ratio; aOR—adjusted odds ratio.

## Data Availability

Data are contained within the article and Supplementary Materials.

## Supplementary Materials

The following supporting information can be downloaded at https://www.mdpi.com/article/10.3390/antibiotics14070625/s1, Figure S1: Clustering profile of M. tuberculosis strains isolated in Sichuan; Table S1: The proposed epidemiological cut-off values (ECOFF/ECVs) for the 12 drugs; Table S2: Genes associated with resistance to the anti-tuberculosis drugs in MTB were identified by WGS.
