# Supplementary material for: Sentinel-Site-Based Surveillance of Mycobacterium tuberculosis Drug Resistance and Epidemiology in Sichuan, China
Source: Antibiotics (Basel). 2025 Jun 20;14(7):625. doi: 10.3390/antibiotics14070625 (PMC12291903; doi:10.3390/antibiotics14070625)
Supplement: Supplementary file 1 [file antibiotics-14-00625-s001.zip › supplementary table.pdf]

Table S1 The proposed epidemiological cut-off values (ECOFF/ECVs) for the 12 drugs

Drugs	Epidemiological cut-off values
Bedaquiline	0.25
Delamanid	0.12
Linezolid	1
Clofazimine	0.25
Isoniazid	0.1
Rifampicin	0.5
Ethambutol	4
Kanamycin	4
Amikacin	1
Ethionamide	4
Moxifloxacin	1
Levofloxacin	1

Table S2 Genes associated with resistance to the anti-tuberculosis drugs in MTB were identified by WGS

Drugs	Mutation type	Number
Rifampicin	rpoB_p.Asp435Glu, rpoB_p.Ser441Leu	1
	rpoB_p.Asp435Val	1
	rpoB_p.His445Asp	1
	rpoB_p.His445Tyr	1
	rpoB_p.His445Tyr, rpoB_p.Glu460Gly	1
	rpoB_p.Ile491Phe	1
	rpoB_p.Leu430Pro	2
	rpoB_p.Leu452Pro	2
	rpoB_p.Phe424Val, rpoB_p.Asp435Glu, rpoB_p.Leu430Pro, rpoB_p.Ser441Leu	1
	rpoB_p.Pro454His	2
	rpoB_p.Ser450Leu	10
	rpoB_p.Ser450Val	1
	rpoB_p.Thr400Ala, rpoB_p.Ser450Leu	2
Isoniazid	ahpC_c.-48G>A	1
	ahpC_c.-52C>T, katG_p.Thr394Ala	1
	ahpC_c.-54C>T, katG_c.1144_1145insC	1
	ahpC_c.-74G>A, fabG1_c.-15C>T	1
	fabG1_c.-15C>T	2
	fabG1_c.-8T>A	1
	katG_p.Gln127Pro, fabG1_c.-15C>T	1
	katG_p.Ser315Asn	2

	katG_p.Ser315Thr	28
	katG_p.Ser315Thr, fabG1_c.-8T>C	2
	katG_p.Ser315Thr, katG_p.Ile335Thr	1
Ethambutol	embA_c.-12C>T	2
	embA_c.-16C>T	1
	embB_p.Asp311Gly, embB_p.Met482Ile	1
	embB_p.Gly406Ala	3
	embB_p.Gly406Asp	1
	embB_p.Gly406Ser	3
	embB_p.Met306Ile	4
	embB_p.Met306Ile, embB_p.Asp1024Asn	1
	embB_p.Met306Val	4
	embB_p.Met306Val, embB_p.Asp1024Asn	1
Fluoroquinolones	gyrA_p.Ala90Val	7
	gyrA_p.Ala90Val, gyrA_p.Asp94Gly	1
	gyrA_p.Ala90Val, gyrA_p.Ser91Pro	1
	gyrA_p.Asp94Ala	4
	gyrA_p.Asp94Ala, gyrA_p.Ser91Pro	1
	gyrA_p.Asp94Gly	3
	gyrA_p.Asp94Tyr	2
	gyrB_p.Arg446Cys	1
	gyrB_p.Asp461Asn	1
Aminoglycosides	rrs_r.1401a>g	3
Ethionamide	ethA_c.1242_1242del	1
	ethA_c.1323_1329del	1
	ethA_c.839_839del, fabG1_c.-8T>C	1
	fabG1_c.-15C>T	4
	fabG1_c.-8T>C	1
